# Production results from piglets vaccinated in a field study in Spain with a Type 1 Porcine Respiratory and Reproductive virus modified live vaccine

**DOI:** 10.1186/s40813-016-0038-x

**Published:** 2016-10-01

**Authors:** Guillermo Cano, Marcia Oliveira Cavalcanti, Francois-Xavier Orveillon, Jeremy Kroll, Oliver Gomez-Duran, Alberto Morillo, Christian Kraft

**Affiliations:** 1 Tests and Trials S.L., Monzon, Spain; 2 Boehringer Ingelheim Veterinary Research Center GmbH & Co. KG, Hannover, Germany; 3 Boehringer Ingelheim Animal Health GmbH, Ingelheim, Germany; 4 Boehringer Ingelheim Vetmedica Inc., Ames, IA USA

**Keywords:** PRRSV, Piglets, Vaccination, Average daily weight gain

## Abstract

**Background:**

PRRS is a viral disease of pigs and sows that is one of the most costly to the pig industry worldwide. The disease can be controlled by focusing on different aspects. One of them is the vaccination of piglets, which is more controversial and difficult to manage than the vaccination of sows. However, pig producers could consider a piglet vaccination strategy if it reduces the negative clinical disease and improves zootechnical performance, decreases the probability to be infected and/or reduces the spread of the virus once the vaccinated piglet is infected. The efficacy of a novel PRRS modified live vaccine (Ingelvac PRRSFLEX® EU) was studied in a blinded, side-by-side placebo controlled field study of piglet vaccination including piglets weaned for three consecutive weeks (week groups 1, 2 and 3).

**Results:**

This study established that PRRS piglet vaccination resulted in significantly better weight gain, seen as early as 4 weeks after vaccination, in naturally challenged pigs. Vaccine efficacy was supported by statistically significant increases in Average Daily Weight Gain (ADWG) among week group 3 vaccinated pigs from vaccination to the end of the study and statistically significant increases in bodyweight and ADWG from inclusion to 10 weeks of age in week group 2 vaccinated piglets. However, no differences were noted in week group 1 presumably because more than 30 % of the vaccinated pigs were viremic at the time of vaccination. Furthermore, the proportion of pigs showing any abnormal clinical sign at least once at any of the examination time points was lower in vaccinated pigs than in control pigs. Based on the viremia results (qPCR), early onset of PRRS was detected in this herd. Viremia occurred at the time of vaccination in week group 1 and shortly after vaccination in week groups 2 and 3. Peak wild type PRRSV infection was assumed at 4 weeks post vaccination in all groups based on the number of PRRS positive pigs in the control groups.

**Conclusion:**

This study establishes that vaccination of piglets with Ingelvac PRRSFLEX® EU at 4 weeks of age improves weight gain and reduces the appearance of clinical sings during the growing period, even when the piglets are infected shortly after vaccination.

**Electronic supplementary material:**

The online version of this article (doi:10.1186/s40813-016-0038-x) contains supplementary material, which is available to authorized users.

## Background

Porcine Reproductive and Respiratory Syndrome (PRRS) is a viral disease of pigs and sows that is one of the most costly to the pig industry worldwide. Nieuwenhuis et al. [1] have calculated a decrease of 1.7 pigs sold per sow during the outbreak period in The Netherlands while in North America 1.44 weaned pigs per sow/year were lost to PRRS [2]. Increasing costs of PRRS between 2005 and 2010 were estimated between 3 and 109 € per sow in Europe [1] and at 2.36$ per pig weaned in US [2].

The disease can be controlled by focusing on different management aspects. Different strategies must be taken into account by veterinarians once a herd has become infected and often include: the fast and reliable diagnosis of an outbreak, internal and external biosecurity measures, control of secondary infections and immunization.

Nowadays, live attenuated and inactivated vaccines are available globally. Also strategies of injections with serum containing live PRRS virus (PRRSV), so called Live Virus Inoculation (LVI) have been used to consistently expose the sow herd [3] but neither of them, vaccines or LVI inoculation are considered to have a high efficacy specially when applied to piglets [4, 5]. Due to the high genetic variability of the virus [6, 7] almost all the infections in the field can be considered as heterologous to existing vaccines [8].

The vaccination of piglets is more controversial among European Veterinarians and difficult to manage (timing and compliance) than the vaccination of sows due to the fact that i) if naïve piglets are infected with genotype 1 PRRSV, the clinical signs of the respiratory disease are not always evident [9], ii) because there is frequent interaction with other pathogens which affects the clinical expression of the symptoms [10] and iii) if the proportion of viremic piglets after weaning is high, the time needed to generate an effective immunity is probably longer than the infection time [11].

Piglet vaccination strategies could be taken into account by the producers if i) the vaccination of piglets decreases the probability to be infected and/or ii) if the vaccination of piglets reduces the spread of the virus once the vaccinated piglet is infected.

Average daily gain and mortality are the performance variables most affected by PRRSV status of piglets [2]. Neither feed conversion rate nor the percentage of pigs sold to the primary market are commonly affected by the PRRSV status of piglets in outbreaks with type 1 virus [2] even though in some cases differences in feed conversion rate has been found [12].

The present study was designed to investigate the efficacy of Ingelvac PRRSFLEX® EU vaccine in 4 week old piglets under field conditions to prevent the productive and clinical effects caused by PRRSV. Primary parameters of vaccine efficacy were the productive performance based on the Bodyweight (BW) and the Average Daily Weight Gain (ADWG). Secondary parameters investigated were viremia, serological response, mortality, clinical signs and concomitant treatments.

## Methods

### Animals and experimental design

The study was performed under normal husbandry field conditions according to Good Clinical Practice (GCP VICH GL9) in two treatment groups. The trial was designed as a randomized, blinded and included an unvaccinated negative control group of piglets. The treatment group received a single intramuscular administration of Ingelvac PRRSFLEX® EU vaccine (PRRS 94881 Modified Live Virus (MLV; vaccinated group) at the minimum titer level indicated for use, while the other group received 1 mL of Phosphate Buffered Saline (PBS) intramuscularly as a negative control group (unvaccinated group). The primary efficacy parameter was weight gain and was compared between vaccinated and unvaccinated pigs. Secondary parameters of the study were mortality, viremia, serology and clinical signs.

A total of 1364 commercial crossbreed pigs (healthy by clinical observation) were included in the study at 4 weeks of age and were distributed to two treatment groups: 690 pigs were administered Ingelvac PRRSFLEX® EU (vaccinated pigs) and 674 pigs a PBS solution (unvaccinated pigs). Three replicates of vaccinated and unvaccinated pigs were included in the study in consecutive weeks: 224, 230 and 236 vaccinated pigs were included in the first Week Group (WG 1), the second Week Group (WG 2) and the third Week Group (WG 3), respectively, and 214, 226 and 234 unvaccinated pigs in WG 1, WG 2 and WG 3, respectively. Study pigs were weaned at 3 weeks of age from the same sow farm. Treatment groups were balanced by sex and initial bodyweight within each replication group. The animal phase finished at the end of the fattening period that was considered when the first pig from each replicate group was ready to go to slaughter.

The selected farm had a previous history of PRRS infection with clinical signs in grower-finisher pigs and was confirmed in a herd pre-screening with PRRSV serology and quantitative Polymerase Chain Reaction (qPCR). Positive qPCR samples were sequenced (Open Reading Frame (ORF) 5) to ensure a heterologous field challenge. In addition, animals were tested for *Actinobacillus pleuropneumoniae* (APP), Swine Influenza Virus (SIV) and Porcines Circovirus Type 2 (PCV2). Sows from the breeding herd were vaccinated with a commercial live attenuated PRRSV vaccine; therefore, seropositive pigs born from vaccinated sows were included in the study. Pigs were fed a commercial ration appropriate for their age and weight. Feed and water were available ad libitum.

**Fig. 1:**
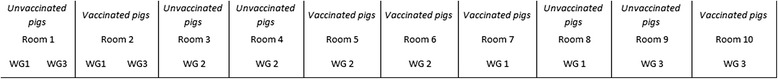
Layout of the pigs at weaning

All study pigs were housed in barns appropriate for their breed and age, and were kept under similar conditions of climate, air quality, ventilation, temperature, air humidity and light. In the post-weaning facilities, pigs from the week groups were distributed in different rooms. WG 1 in rooms 1, 2, 7 and 8; WG 2 in rooms 3, 4, 5 and 6 and WG 3 in rooms 1, 2, 9 and 10 (see Fig. 1). Piglets of different WG in the same room were kept in separate pens. Vaccinated pigs were housed separately from the unvaccinated pigs until entry into fattening. Due to the different sizes of rooms, the randomization of the rooms to the treatment groups was done considering the room effect. Cross-contamination was prevented by strict biosecurity rules implemented on the farm for the duration of the study. In the fattening facilities, vaccinated and unvaccinated pigs were commingled and distributed in three buildings.

Pigs were individually weighed at four time points: at vaccination prior to administration of Ingelvac PRRSFLEX® EU or PBS, at 4 weeks post-vaccination, at the beginning of fattening (10 weeks post-vaccination) and at the end of the study. For WG1 and WG2, the end of the study was at 17 weeks post-vaccination and for WG 3 at 16 weeks post-vaccination. ADWG for the intervals between weighing at vaccination and weighing at 4, 10 and 16–17 weeks post-vaccination were calculated for each pig individually. Homogeneity of the BW at the end of the study was calculated from Coefficient of Variation (1-CV) for every group and within week group.

Mortality was recorded throughout the study for calculation of the mortality rate. Furthermore, study pigs were clinically examined at weeks 4, 10, 14 and 16 or 17 post-vaccination. Special attention was given to the respiratory signs (dyspnea, cough) and apathy, but skin alterations (petechiae, crust, anemia or icterus), joint disorders and diarrhea were also recorded. Collective and individual treatments were also recorded throughout the study.

### Collection and processing of samples

Blood samples were collected from 73 vaccinated (24, 23 and 26 in WG 1, WG 2 and WG 3, respectively) pigs and 69 unvaccinated pigs (22, 24 and 23 in WG 1, WG 2 and WG 3, respectively). In each pen, at least one pig of middle weight was chosen to be bled at vaccination time. Blood samples were drawn prior to administration of Ingelvac PRRSFLEX® EU or PBS. Additional blood samples were collected from the same pigs at 4, 10, 14 and 16 or 17 weeks after vaccination. Blood samples were collected by jugular *venipuncture using 4 mL dry vacuum tubes* and were processed within 24 h after collection in order to obtain serum by centrifugation at 3000 rpm during 10 min. Serum samples were stored frozen at −80 °C until the end of the study. Then, the serum samples were sent to Boehringer Ingelheim Veterinary Research Center GmbH & Co. KG to be tested for PRRS antibodies by ELISA (HerdChek PRRS X3 Antibody Test Kit,IDEXX Laboratories, Inc.), and detection of PRRSV-EU specific RNA via real-time reverse transcription PCR. Proportions of positive animals were calculated per time point of examination.

### Vaccine and placebo product description

Piglets were vaccinated intramuscularly in the neck with one dose (1 ml) of Ingelvac PRRSFLEX® EU vaccine with a minimum titer as indicated on the vaccine label instructions at 4-weeks of age. Control animals were administered one dose of vaccine corresponding solvent (PBS) without vaccine content. No other vaccinations or treatments were administered to the animals on at least 3 days before and after the PRRS vaccine treatment.

### Data analysis

Statistical analysis was performed with R software [13]. All tests were designed as two-sided tests and differences were considered as statistically significant if *p* ≤ 0.05 For BW and ADWG, differences between treatment groups were tested using analysis of variance and subsequent t-tests. Treatment group (PRRS 94881 MLV or PBS), week group (WG 1, WG 2 or WG 3), their interaction (Group*WG) and sex (male or female) were included as factors in the statistical model. The initial weight (BW at vaccination time) was used as covariate for all post-treatment time points and for all periods. Least squares means of the groups and differences between least squares means with 95 % Confidence Intervals (95 % CI) were calculated from the analysis of variance. Homogeneity at final BW was tested comparing variances of BW at the end of the study using a Fisher’s test. Differences in proportions (qPCR positive, ELISA positive, mortality rate, clinical observations and concomitant treatments) between the treatment groups were tested by Fisher’s exact test. Wilson’s confidence interval for a single proportion was also calculated for every proportion.

## Results

The farm showed a high degree of PRRSV positive animals in a pre-screening before the study started. Positive qPCR samples were sequenced (ORF 5, see Additional file 1) and results showed identities of 88.94, 88.45 and 92.74 % to Lelystad virus (GenBank Accession Number: M96262), Porcilis® PRRS (the PRRSV isolate of the commercial live attenuated PRRS virus vaccine used in sows; GenBank Accession Number: KJ127878) and Ingelvac PRRSFLEX® EU (GenBank Accession Number: KT988004), respectively. Moreover, the pre-screening revealed pigs were positive for APP, SIV and PCV2 antibodies.

BW and ADWG from vaccinated and unvaccinated pigs are shown in Table 1. Vaccinated pigs showed better growth parameters than unvaccinated pigs at 10 weeks after vaccination. Nevertheless, looking at the 3 week groups separately, there were differences (*p* < 0.05) between vaccinated and unvaccinated pigs in WG 2 and WG 3 but not in WG 1 (Table 1).

**Table 1:** Bodyweight (BW) and average daily weight gain (ADWG) at different observation periods. Least square mean ± standard error

Parameter	Week Group 1 + 2 + 3		Week Group 1		Week Group 2		Week Group 3	
	Unvaccinated pigs	Vaccinated pigs	Unvaccinated pigs	Vaccinated pigs	Unvaccinated pigs	Vaccinated pigs	Unvaccinated pigs	Vaccinated pigs
Number of animals	674	690	214	224	226	230	234	236
BW (kg) at								
Vaccination	5.8 ± 0.05	5.8 ± 0.05	5.8 ± 0.09	5.7 ± 0.09	5.7 ± 0.09	5.7 ± 0.09	6.1 ± 0.09	6.1 ± 0.09
4 weeks post-vaccination	14.6 ± 0.10	14.7 ± 0.10	14.4 ± 0.17	14.1 ± 0.17	15.1^a^ ± 0.17	15.6^b^ ± 0.17	14.2 ± 0.17	14.3 ± 0.16
10 weeks post-vaccination	40.9^a^ ± 0.23	41.5^b^ ± 0.23	40.0 ± 0.41	39.4 ± 0.40	43.3^a^ ± 0.42	44.9^b^ ± 0.41	39.3 ± 0.40	40.4 ± 0.40
16–17 weeks post-vaccination†	76.6 ± 0.37	76.9 ± 0.37	77.1 ± 0.65	76.4 ± 0.64	81.3 ± 0.66	80.9 ± 0.64	71.3^a^ ± 0.64	73.4^b^ ± 0.63
ADWG (g/d) from vaccination to								
4 weeks post-vaccination	310 ± 3.5	314 ± 3.4	306 ± 6.1	295 ± 6.0	328^a^ ± 6.1	347^b^ ± 6.0	296 ± 5.9	300 ± 5.9
10 weeks post-vaccination	486^a^ ± 3.3	495^b^ ± 3.2	474 ± 5.7	465 ± 5.6	519^a^ ± 5.8	542^b^ ± 5.7	465 ± 5.5	479 ± 5.5
16–17 weeks post-vaccination†	602 ± 3.2	605 ± 3.1	597 ± 5.6	591 ± 5.5	638 ± 5.6	635 ± 5.5	573^a^ ± 5.4	590^b^ ± 5.4
BW homogeneity‡ (%) at								
16–17 weeks post-vaccination†	85^a^	86^b^	86	86	87	87	82^a^	85^b^

*BW* Bodyweight, *ADWG* Average Daily Weight Gain

† The end of the study was at 17 weeks post-vaccination in week groups 1 and 2, and at 16 weeks post-vaccination in week group 3

‡ BW homogeneity was calculated from coefficient of variation (1-CV). It was tested comparing variances of BW using an F test

^a, b^ Within week group, different letter in the same row indicates statistically significant difference (*p* < 0.05). Differences between treatment groups were tested using analysis of variance and subsequent t-tests

The BW of vaccinated pigs from WG 2 at 4 and 10 weeks after vaccination was higher (*p* < 0.05) than unvaccinated pigs within the same WG. ADGW increased from vaccination to 4 and 10 weeks after vaccination (*p* < 0.05; Table 1).

The BW in WG 3 of vaccinated pigs was higher (*p* < 0.05) at week 16 after vaccination and ADWG increased from vaccination to 16 weeks after vaccination (*p* < 0.05; Table 1).

The BW uniformity at the end of fattening was better in vaccinated pigs than in unvaccinated (*p* < 0.05; Table 1). Taking into account week groups separately, the BW uniformity of vaccinated pigs improved only in WG 3 (*p* < 0.05; Table 1) and there were not differences between groups (*p* > 0.05) in WG 1 and WG 2.

**Fig. 2:**
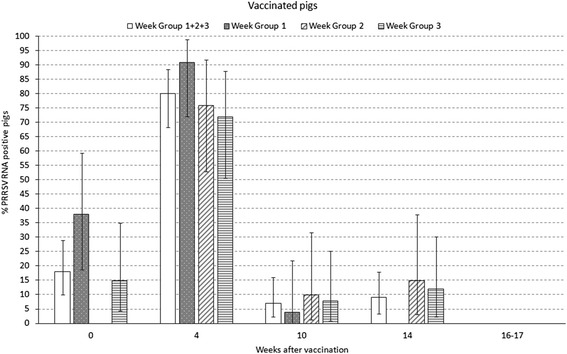
Viremia in % positive pigs (qualitative) by qPCR at the different sampling times in 73 vaccinated pigs (24, 23 and 26 in week groups 1, 2 and 3, respectively). Percentage of positive pigs and confidence interval 95 %

**Fig. 3:**
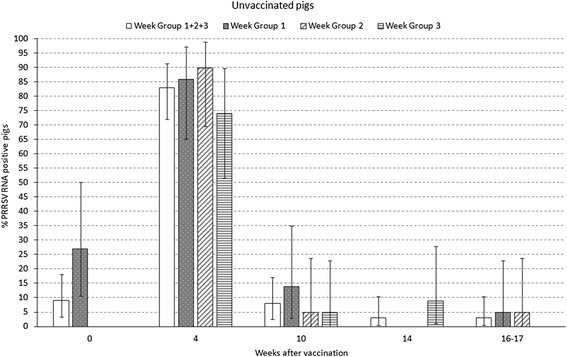
Viremia in % positive pigs (qualitative) by qPCR at the different sampling times in 69 unvaccinated pigs (22, 24 and 23 in weeks groups 1, 2 and 3, respectively). Percentage of positive pigs and confidence interval 95 %

Percentage of PRRSV RNA positive pigs by qPCR throughout the study is shown in Figs. 2 and 3 for vaccinated and unvaccinated pigs. Peak of viremia with 80 % (68–88; 95 % CI) of vaccinated pigs and 83 % (72–91 %; 95 % CI) of unvaccinated pigs tested positive was at 4 weeks after vaccination. For all the time points, no differences were observed in the prevalence of viremic animals between vaccinated and unvaccinated pigs. For pigs in WG1, the PRRSV field infection was on-going at vaccination time with 38 % (19–59 %; 95 % CI) of vaccinated pigs and 27 % (11–50 %; 95 % CI) of unvaccinated pigs tested positive (see Figs. 2 and 3 at week 0). Notably, none of the vaccinated animals tested positive by PRRSV qPCR at the end of fattening while 3 % of the unvaccinated animals were still viremic at the end of fattening.

Percentages of pigs detected serologically positive by ELISA were determined in vaccinated and unvaccinated pigs. It was observed that 67 % (55–77 %; 95 % CI) of vaccinated pigs and 75 % (63–85 %; 95 % CI) of unvaccinated pigs were seropositive at vaccination, and that 99 % (92–100 %; 95 % CI) of vaccinated pigs and 92 % (83–98 %; 95 % CI) of unvaccinated pigs were already seropositive at 4 weeks post-vaccination. For all time points within this study, there was not statistical difference in the seroconversion rate between vaccinated and control pigs.

Since the wild type PRRSV infection took place very early during (WG 1) or after (WG 2 and WG 3) vaccination, it was not possible to determine the source of viremia in vaccinated animals.

Table 2 summarizes the mortality, clinical signs and concomitant treatments throughout the study. Proportion of pigs showing any abnormal clinical sign at least once at any of the examination time points was lower (*p* < 0.05) in vaccinated pigs than in unvaccinated pigs, being the highest difference in WG 3 pigs. The most frequent signs were respiratory and skin alterations, being in both instance the proportion of affected pigs lower (*p* < 0.05) in vaccinated pigs than in unvaccinated (*p* < 0.05). Prevalence of joint disorders and diarrhea was generally low (1 % or lower).

**Table 2:** Percentage of mortality, pigs with clinical signs and pigs received concomitant treatments throughout the study (95 % CI)

Parameter	Week Group 1 + 2 + 3		Week Group 1		Week Group 2		Week Group 3	
	Unvaccinated pigs	Vaccinated pigs	Unvaccinated pigs	Vaccinated pigs	Unvaccinated pigs	Vaccinated pigs	Unvaccinated pigs	Vaccinated pigs
Number of animals	674	690	214	224	226	230	234	236
Mortality (%)	6.1	4.9	5.1	3.6	5.8	4.8	7.3	6.4
	(4.5–8.2)	(3.6–6.8)	(2.9–9.0)	(1.8–6.9)	(3.4–9.6)	(2.7–8.4)	(4.6–11.3)	(3.9–10.2)
Any clinical sign (%)†	8.3^a^	4.2^b^	7.0	3.6	6.2	3.0	11.5^a^	5.9^b^
	(6.5–10.6)	(2.9–6.0)	(4.3–11.2)	(1.8–6.9)	(3.7–10.1)	(1.5–6.2)	(8.1–16.3)	(3.6–9.7)
Respiratory signs (%)	4.7^a^	2.3^b^	3.3	1.8	3.1	1.3	7.7	3.8‡
	(3.4–6.6)	(1.4–3.7)	(1.6–6.6)	(0.7–4.5)	(1.5–6.3)	(0.4–3.8)	(4.9–11.8)	(2.0–7.1)
Skin alterations (%)	2.4^a^	0.7^b^	2.3	0.9	1.3	0.4	3.4	0.8
	(1.5–3.8)	(0.3–1.7)	(1.0–5.4)	(0.3–3.2)	(0.5–3.8)	(0.8–2.4)	(1.7–6.6)	(0.2–3.0)
Concomitant treatments (%)	23.0^a^	18.6^b^	15.5	15.6	21.2^a^	12.2^b^	32.5	27.5
	(20.0–26.3)	(15.8–21.6)	(10.4–19.8)	(11.5–21.0)	(16.4–27.0)	(8.6–17.0)	(26.8–38.7)	(22.2–33.6)

95 % CI: Wilson’s Confidence Interval 95 % for a single proportion

† Respiratory signs and/or skin alterations and/or joint disorders and/or diarrhoea. Prevalence of joint disorders and diarrhoea was 1 % or lower

^a, b^ Within week group, different letter in the same row indicates statistically significant difference (*p* < 0.05). Differences between the treatment groups were tested by Fisher’s exact test

Regarding concomitant treatments (Table 2), the proportion of vaccinated pigs treated individually at least once with a parenteral treatment was lower than in unvaccinated pigs (*p* < 0.05). The highest difference between vaccinated and unvaccinated pigs was in WG 2. About 98 % of the individual treatments administered were with injectable Enrofloxacin due to respiratory signs and in some cases due to diarrhea. Although collective treatments were restricted, a treatment with Doxycycline in water was administered to all piglets for 6 days once. The whole population was medicated including vaccinates and controls but as pigs of different ages were treated this occurred at a different time point in relation to the day of vaccination. Medication was at 4, 3 and 2 weeks post-vaccination in WG 1, WG 2 and WG 3, respectively. This treatment was administered due to respiratory signs (sneezing and coughing), but also rough haircoat and thinness observed affecting all nursery rooms on the farm.

While numerically lower, there was no difference in mortality rate between vaccinated and unvaccinated pigs neither in the overall study nor within replicate groups (Table 2). None of the dead pigs examined in both treatment groups revealed PRRS related gross necropsy findings. The incidence of the findings recorded did not provide indications of a treatment-related pattern.

## Discussion

This large field study was performed to investigate the efficacy of a novel PRRS MLV vaccine in terms of zootechnical parameters (BW and ADGW), in piglets of 4 weeks of age that originated from a vaccinated sow farm undergoing an active infection with a wild type PRRSV.

Vaccinated pigs showed better growth parameters than unvaccinated pigs at 10 weeks after vaccination. This performance improvement was observed in the critical period of virus circulation supporting the efficacy of the vaccine. When analyzing the data by separate cohorts it is clear that there were different responses to vaccination. Statistically significant differences for BW and ADWG at the end of the study were observed only in WG 3 pigs. Very few well controlled PRRS field trials have been conducted in Europe. The differences in BW and in ADWG observed among treatment groups in this study were not as remarkable as in previous reports [14–17]. Probably, this is due to the short time between the vaccination and the occurrence of the natural infection, which did not allow for better performance in vaccinated animals. In particular, the improvement in BW and ADWG could not be demonstrated in WG 1 probably because of a PRRSV wild type infection at the time of vaccination. This was confirmed through qPCR testing of pig sera at study day −1 before vaccination which showed 33 % PRRSV RNA pigs. In addition most of those previous studies were conducted in PRRS genotype 2 outbreaks under US conditions.

Proper vaccination against PRRSV or any MLV vaccines involves immunization of healthy pigs and proper timing of the vaccination event in relationship to onset of disease pressure on the farm. Under field conditions, the onset of immunity could be adversely affected by the presence of confounding factors, including the presence of other pathogens and vaccination in the face of an ongoing active PRRSV infection [14].

The interpretation of these results has to be done considering these circumstances; however, it seems that the vaccine protection even in the face of an existing infection was able to result in differences in BW and ADGW at 10 weeks of age. As the end of the study was set as the time when the first batch of pigs was sent to the slaughterhouse, we do not know if these differences carry over to batch closeout, where the entire economic impact is assessed by the producers.

Respiratory clinical signs, skin alterations and concomitant treatments were all found to be significantly reduced in vaccinated animals compared to unvaccinated animals suggesting a beneficial effect of vaccination on secondary infections and the general health status of the animals. Mortality was not statistically different between vaccinated and control pigs. This and other clinical parameters could be affected by the medication with Doxycycline to the population.

None of the necropsies performed during the study showed macroscopic signs of PRRSV infection, but detailed diagnostics of each case were not performed. While mortality was somewhat elevated at 4.9 and 6.1 % (vaccinated vs. control, respectively) it was lower than in other field studies that demonstrated statistical differences in reduction of mortality after vaccination [15]. We can assess that mortality was caused mainly by secondary infections in this trial (data not shown) as has been the case of other studies [18]. A more detailed diagnostic investigation of the deaths in this trial could help explain the lack of statistical reduction in mortality.

At inclusion in the study at 4 weeks of age most animals were serologically positive. This might be due to maternally derived antibodies from vaccinated dams or already signals the ongoing field infection that started during the suckling period. As vaccination is recommended from 17 days of life onwards, maternally derived antibodies should not have interfered with vaccination and were not likely providing protection as many were also positive for viral RNA. A limitation of this field study, since the wild type PRRSV infection took place very soon after vaccination or even before vaccination, was that it was not possible to determine the source of viremia in these animals. qPCR positive serum samples could be due to vaccine virus, the field virus (being viremic at vaccination time) or both as reported in other studies [11]. Sequencing of all positive samples may have provided further data to clarify this issue. Early PRRSV infection in this trial highlights the importance of ensuring breeding herd stability, defined by consistently weaning PRRSV negative piglets, to maximize the benefit of piglet vaccination [4, 17]. Other studies from North America have demonstrated that the direct benefits of PRRS vaccination in terms of efficacy depend on vaccination ahead of infection with field virus [14].

The PRRS field virus in this trial can be considered heterologous to the vaccine virus (92.74 % homology in ORF5). Murtaugh [19] indicates that a homology less or equal than 97–98 % can be considered as a different strain of the PRRSV, although the scientific community has not agreed on a defined cut off. Even though significant genetic differences were found between the strain circulating before vaccination and the vaccine strain, the vaccine provided partial clinical protection. However, to better understand the dynamics of the infection and the protection of the vaccine it would be advisable in future studies to perform such type of analyses.

## Conclusions

This study establishes that vaccination of piglets with Ingelvac PRRSFLEX® EU at 4 weeks of age improves weight gain and reduces the appearance of clinical sings during the growing period, even when the piglets are infected shortly after vaccination. Evidence of vaccine benefits under field conditions was provided by improved performance in the period during the onset and peak viremia of wild-type PRRSV.

## Abbreviations

ADWG, average daily weight gain; AEMPS, Agencia Española de Medicamentos y Productos Sanitarios; APP, Actinobacillus pleuropneumoniae; BW, bodyweight; CI, confidence interval; CV, coefficient of variation; LVI, live virus inoculation; MLV, modified live vaccine; ORF, open reading frame; PBS, phosphate buffered saline; PCV2, Porcines Circovirus Type 2; PRRS, Porcine Reproductive and Respiratory Syndrome; PRRSV, PRRS virus; qPCR, quantitative Polymerase Chain Reaction; RNA, ribonucleic acid; SIV, Swine Influenza Virus; WG, weight group.

## Additional file

Additional file 1: ORF5 sequence of the PRRSv field strain. (TXT 641 bytes)

## References

[1] Nieuwenhuis N, Duinhof TF, van Nes A (2012). Economic analysis of outbreaks of porcine reproductive and respiratory syndrome virus in nine sow herds. Vet Rec.

[2] Holtkamp DJ, Kliebenstein JB, Neumann EJ, Zimmerman JJ, Rotto HF, Yoder TK (2013). Assessment of the economic impact of porcine reproductive and respiratory syndrome virus on United States pork producers. J Swine Health Prod.

[3] Fano E, Olea L, Pijoan C (2005). Eradication of porcine reproductive and respiratory syndrome virus by serum inoculation of naive gilts. Can J Vet Res.

[4] Kimman TG, Cornelissen LA, Moormann RJ, Rebel JMJ, Stockhofe-Zurwieden N (2009). Challenges for porcine reproductive and respiratory syndrome virus (PRRSV) vaccinology. Vaccine.

[5] Linhares DCL, Cano JP, Torremorell M, Morrison RB (2014). Comparison of time to PRRSV-stability and production losses between two exposure programs to control PRRSV in sow herds. Prev Vet Med.

[6] Mateu E, Martín M, Vidal D (2003). Genetic diversity and phylogenetic analysis of glycoprotein 5 of European-type porcine reproductive and respiratory virus strains in Spain. J Gen Virol.

[7] Murtaugh MP, Stadejek T, Abrahante JE, Lam TTY, Leung FC-C (2010). The ever-expanding diversity of porcine reproductive and respiratory syndrome virus. Virus Res.

[8] Pileri E, Gibert E, Soldevila F, García-Saenz A, Pujols J, Diaz I (2015). Vaccination with a genotype 1 modified live vaccine against porcine reproductive and respiratory syndrome virus significantly reduces viremia, viral shedding and transmission of the virus in a quasi-natural experimental model. Vet Microbiol.

[9] Martínez-Lobo FJ, Díez-Fuertes F, Segalés J, García-Artiga C, Simarro I, Castro JM (2011). Comparative pathogenicity of type 1 and type 2 isolates of porcine reproductive and respiratory syndrome virus (PRRSV) in a young pig infection model. Vet Microbiol.

[10] Van Gucht S, Labarque G, Van Reeth K (2004). The combination of PRRS virus and bacterial endotoxin as a model for multifactorial respiratory disease in pigs. Vet Immunol Immunopathol.

[11] Labrecque MP, Cardinal F (2013). Impact of PRRS vaccination timing in pigs with different maternal immunity levels. Proceedings of the 44th Annual Meeting of the American Association of Swine Veterinarians,San Diego, CA, USA.

[12] Kritas SK, Alexopoulos C, Kyriakis CS, Tzika E, Kyriakis SC (2007). Performance of fattening pigs in a farm infected with both porcine reproductive and respiratory syndrome (PRRS) virus and porcine circovirus type 2 following sow and piglet vaccination with an attenuated PRRS vaccine. J Vet Med Ser Physiol Pathol Clin Med.

[13] R Core Team. R: A language and environment for statistical computing Vienna, Austria. [Internet]. Vienna, Austria: R Foundation or Statistical Computing; 2014. Available from: http://www.R-project.org/

[14] Philips RC, Edler RA, Holck JT (2006). Vaccination with MLV vaccine to control PRRS in growing pigs. Proceedings of the 19th International Pig Veterinary Society Congress, Copenhagen, Denmark.

[15] Polson D, Baker RB, Philips RC, Hotze B (2008). Improved growing pig performance in a large production system applying intensive management and vaccination protocol. Proceedings of the 20th International Pig Veterinary Society Congress, Durban, South Africa.

[16] Angulo J, Philips RC, Cano JP (2010). Growth peformance improvement and mortality reduction derived from a PRRS large scale control project in the US. Proceedings of the 21st International Pig Veterinary Society Congress, Vancouver, Canada.

[17] Robbins R, Harms P, Angulo J, Scheidt A, Philips RC, Kolb J (2013). PRRSV control in finisher pigs, a large scale barn study in high dense area in USA. Proceedings of the 44th Annual Meeting of the American Association of Swine Veterinarians, San Diego, CA, USA.

[18] Trus I, Bonckaert C, van der Meulen K, Nauwynck HJ (2014). Efficacy of an attenuated European subtype 1 porcine reproductive and respiratory syndrome virus (PRRSV) vaccine in pigs upon challenge with the East European subtype 3 PRRSV strain Lena. Vaccine.

[19] Murtaugh M (2012). Use and interpretation of sequencing in PRRSV control programs. Proceedings of the 2012 Allen D. Leman Swine Conference, St Paul, MN, USA.

